# Beyond the Mask―Psychological Discomfort as a Predictor of Early CPAP Nonadherence in Moderate‐to‐Severe OSA Patients: A Prospective Mixed‐Methods Study

**DOI:** 10.1155/nrp/9630236

**Published:** 2026-04-06

**Authors:** Yen-Chin Chen, Cristina Frange, Shin-Shan Pan, Cheng-Man Ng, Yen-Hsu Chen, Cheng-Yu Lin

**Affiliations:** ^1^ Department of Nursing, College of Medicine, National Sun Yat-sen University, Taiwan/ No.70 Lien-hai Road, Kaohsiung, 804201, Taiwan, nsysu.edu.tw; ^2^ Department of Nursing, College of Medicine, National Cheng Kung University, Taiwan/ No. 1, Dasyue Rd, East District, Tainan City, 701, Taiwan, ncku.edu.tw; ^3^ School of Nursing, College of Nursing, Kaohsiung Medical University, Taiwan/ Shih-Chuan 1st Road, Kaohsiung, 80708, Taiwan, kmu.edu.tw; ^4^ Sleep Health Physiotherapy Center, Brazil/Avenida Onze de Junho, 1070, Conjunto 510, Vila Clementino, São Paulo, Brazil; ^5^ Department of Otolaryngology, College of Medicine, National Cheng Kung University Hospital, National Cheng Kung University, Taiwan/ No.138, Sheng Li Road, North Dist., Tainan City, 704, Taiwan, ncku.edu.tw; ^6^ Division of Infectious Diseases, Department of Internal Medicine, Kaohsiung Medical University Hospital, Kaohsiung Medical University, Kaohsiung, Taiwan, kmu.edu.tw; ^7^ School of Medicine, Graduate Institute of Medicine, Sepsis Research Center, Center of Tropical Medicine and Infectious Diseases, Kaohsiung Medical University, Kaohsiung, Taiwan, kmu.edu.tw; ^8^ Department of Biological Science and Technology, College of Biological Science and Technology, National Chiao Tung University, Hsinchu, Taiwan, nctu.edu.tw; ^9^ Sleep Medicine Center, College of Medicine, National Cheng Kung University Hospital, National Cheng Kung University, Taiwan// No.138, Sheng Li Road, North Dist., Tainan City, 704, Taiwan, ncku.edu.tw

**Keywords:** adherence, continuous positive airway pressure (CPAP), mixed-methods research, obstructive sleep apnea (OSA), symptoms

## Abstract

**Background and Objective:**

Early adherence to CPAP therapy is a critical determinant of long‐term treatment adherence, but it is frequently compromised by patient‐reported adverse effects. This study aimed to systematically identify CPAP‐related adverse symptoms and evaluate their associations with early treatment adherence among patients with moderate‐to‐severe OSA.

**Methods:**

A prospective mixed‐methods study was conducted at a tertiary sleep center between August 2021 and February 2025. A total of 121 adults with newly diagnosed moderate‐to‐severe OSA were enrolled. CPAP‐related symptoms (*n* = 430) were collected through semistructured telephone interviews conducted at least three times. Symptoms were categorized into four domains: physiological, psychological, CPAP interface–related, and CPAP device–related. Early CPAP adherence over a 14‐day trial period was defined as CPAP use for ≥ 4 h per night on ≥ 70% of nights.

**Results:**

Among 121 participants (mean age 50.8 years; 84.3% male), 60.3% demonstrated good adherence. Psychological symptoms were the only domain significantly associated with reduced adherence (*β* = −7.50%; *p* = 0.049), with suffocation sensations showing a particularly strong negative impact (*β* = −37.14%; *p* = 0.018). Conversely, CPAP interface–related symptoms such as mask discomfort were positively associated with adherence (*β* = 12.89%; *p* = 0.011). Physiological and equipment‐related symptoms were not significantly associated with CPAP adherence.

**Conclusion:**

Psychological discomfort significantly impairs early CPAP adherence among patients with OSA. Routine assessment and timely intervention targeting psychological and interface‐related complaints during the initial treatment period may help support continued CPAP use in clinical practice.

**Trail Registration:** ClinicalTrials.gov_identifier: NCT06520592

**Summary**
 What was the test?
◦ This study examined the association between CPAP‐related adverse symptoms and early treatment adherence among patients with moderate‐to‐severe OSA.
• What the study showed?
◦ Psychological discomfort, especially suffocation sensations, was significantly associated with reduced initial CPAP adherence.
• Clinical significance:
◦ These findings underscore the importance of early psychosocial assessment and targeted symptom‐guided interventions to support sustained CPAP use in clinical practice.

## 1. Introduction

Obstructive sleep apnea (OSA) is characterized by frequent upper airway collapse, leading to obstructive apnea, intermittent hypoxia, and hypercapnia. These physiological disturbances result in fragmented nocturnal sleep, reductions in oxygen saturation, and excessive daytime sleepiness [1, 2]. Continuous positive airway pressure (CPAP) is the first‐line noninvasive treatment for moderate‐to‐severe OSA and remains the most effective intervention [3]. Its mechanism involves delivering CPAP to maintain upper airway patency, thereby reducing hypoxia‐induced arousal during sleep [4].

Although CPAP is the first‐line prescription for treating OSA, its therapeutic effectiveness is often compromised due to poor adherence. Adherence to CPAP is commonly defined as the use of CPAP for more than 4 h per night on at least 70% of nights [5]. The factors influencing adherence to CPAP use are complex and multifaceted. A critical literature review by Ghadiri and Grunstein highlighted a wide range of CPAP‐related side effects (e.g., mask leakage, skin irritation, facial pressure), as well as psychological and device‐related factors such as claustrophobia, infection, or device noise [6]. Park et al. also identified physiological discomfort as a key factor contributing to CPAP nonadherence [7]. These discomforts often result in difficulties falling asleep and reduced sleep quality, ultimately leading to significantly lower adherence rates [8].

Previous studies employing various methodologies and outcome measures have reported a wide range of CPAP‐related side effects; however, few have adopted a mixed‐methods design grounded in patient‐centered care or addressed this heterogeneity through a structured, thematic framework. In addition, early adherence to CPAP therapy is strongly correlated with long‐term adherence [9]. This underscores the clinical value of assessing common adverse effects at the onset of therapy to identify individuals at risk for poor adherence. Therefore, the present study aims to investigate the prevalent adverse symptoms experienced by patients with moderate‐to‐severe OSA during initial CPAP use and to determine which symptoms are associated with reduced adherence.

## 2. Methods

### 2.1. Study Design and Setting

This prospective observational study employed an explanatory sequential mixed‐methods design, in which qualitative data were collected at three time points during CPAP initiation: day 3, day 7, and between day 13 and day 15 (corresponding to one day prior to the scheduled 2‐week clinic follow‐up visit), and analysis preceded quantitative modeling. Integration occurred through a qualitative‐to‐quantitative data transformation process, whereby patient‐reported experiences were systematically analyzed and converted into symptom domains. These domains were subsequently refined and validated through expert consensus, forming the core integrative component of the mixed‐methods design, consistent with established mixed‐methods methodology literature [10, 11]. Qualitative data were used to identify and operationalize symptom domains that were subsequently tested in quantitative models. The resulting symptom variables were then examined in quantitative models to assess their associations with CPAP adherence. This study was reported in accordance with the Strengthening the Reporting of Observational Studies in Epidemiology (STROBE) guidelines (see Supporting file S1).

### 2.2. Study Setting

The study was conducted at a sleep specialist clinic within a medical center from August 2021 to February 2025. In the general sleep medicine clinic, individuals presenting with sleep‐related symptoms, such as excessive daytime sleepiness, loud snoring, or observed nocturnal apneas, are referred by sleep specialists for a sleep study. If the PSG results confirm moderate and severe OSA (defined as an apnea‒hypopnea index (AHI) ≥ 15 events per hour), physicians typically recommend CPAP therapy as the first‐line treatment. At this clinic, patients are offered a complimentary free CPAP trial for a period of 2 weeks.

### 2.3. Participants

We included patients aged ≥ 20 years because this age threshold corresponds to the legal definition of adulthood under Taiwan’s Civil Code. Eligible participants were diagnosed with moderate‐to‐severe OSA (AHI ≥ 15) confirmed via polysomnography (PSG). Patients with a history of CPAP use, central sleep apnea, referrals from other institutions, those planning to undergo oropharyngeal surgery during the study period, patients with uncontrolled acute psychiatric disorders, or those with terminal illnesses were excluded.

Although the study was conducted at a high‐volume tertiary sleep center with six‐bed sleep laboratory, enrollment was intentionally restricted to a narrowly defined clinical population. Many patients were excluded due to prior CPAP use, preference for alternative treatments, inability to participate in structured follow‐up, or medical and psychiatric exclusion criteria. As a result, the final sample of 121 participants represents a purposefully selected cohort appropriate for evaluating early CPAP‐related symptoms and adherence over a 3.5‐year study period. Details of participant recruitment and follow‐up have been described in our previous study [12].

The statistical power was estimated using G ∗ Power version 3.1.9.2. Based on findings from our previous study [12], which demonstrated a significant association between improvements in daytime sleepiness and CPAP adherence, we used this relationship to inform an approximate effect size. Assuming a small‐to‐moderate effect size (Cohen’s *f* ^2^ ≈ 0.20) for a key predictor, informed by prior generalized estimating equations (GEEs) results (*B* = −0.02), a sample size of 121 participants would provide approximately 88% power at a two‐sided *α* level of 0.05 to detect an association of this magnitude under a repeated‐measures framework.

### 2.4. Outcome Variables

CPAP adherence data were obtained after participants provided written informed consent authorizing ResMed to access their CPAP cloud‐based usage data. CPAP usage data were collected from the ResMed AirSense 10 device and included 14 days of objective CPAP use records.

CPAP adherence calculated using the following formula:

 Adherence (%) = Total days with CPAP usage ≥ 4 h × 100%.
 Good adherence to CPAP is defined as the use of CPAP for ≥ 4 h per night for at least 70% of the total nights [5].

### 2.5. Covariates

#### 2.5.1. Symptoms of CPAP Use During the 2 Week of Therapy

Symptoms associated with CPAP use were collected through repeated semistructured telephone interviews conducted by a single certified sleep care manager (Shih‐Shan Pan) during the early phase of therapy. When a participant reported the same symptom more than once, it was counted only once at the observation level. However, when participants reported different symptoms, each symptom was recorded separately. This short period of intensive symptom monitoring was not only a strong predictor of CPAP adherence [13] but was also designed to be feasible within routine clinical practice. Interviews were carried out during routine working hours (09:00–18:00) and lasted approximately 15–20 min per call. A standardized semistructured interview guide was used to explore CPAP‐related discomfort, concerns, and adverse experiences through open‐ended questions, with additional probing as needed (see Supporting File S2).

The classification of CPAP‐related symptoms into different domains was theoretically informed by the framework proposed by Ghadiri and Grunstein [6], which conceptualizes CPAP side effects based on a critical review of the literature. Patient‐reported symptoms were first elicited through open‐ended interviews and subsequently categorized using content analysis guided by this framework. Two researchers (YC Chen and CY Lin) independently reviewed interview records, identified symptom expressions, and coded them into preliminary categories. Discrepancies were resolved through discussion to achieve consensus. Rather than generating narrative themes for interpretive purposes, the qualitative analysis was conducted with the specific aim of comprehensively identifying and categorizing patient‐reported symptoms relevant to CPAP adherence.

To enhance clinical validity and reliability, the resulting symptom classification framework was further refined and validated using a Delphi consensus process involving seven sleep experts from different disciplines, including sleep physicians, technologists, and sleep care managers. Following expert consensus, each distinct symptom category was operationalized as a binary variable (0 = not reported; 1 = reported at least once during the 2‐week CPAP trial period). Domain‐ and subdomain‐level variables were constructed by aggregating related symptom categories in accordance with the framework proposed by Ghadiri and Grunstein. Accordingly, higher percentages reflected a greater frequency of symptom occurrence rather than symptom severity.

#### 2.5.2. Demographic Data

The collected demographic data included age, gender, educational level, comorbidities (e.g., hypertension, diabetes, cardiovascular disease, chronic obstructive pulmonary disease, asthma, and cancer), smoking status (yes/no), alcohol consumption (yes/no), exercise habits (yes/no), and afternoon napping habits. Clinical data included self‐reported daytime sleepiness assessed using the Epworth Sleepiness Scale (ESS) and PSG parameters, including AHI.

### 2.6. Data Collection Procedure

This study was approved by the institutional ethics committee (Approval No. A‐ER‐111–531) and conducted in accordance with all relevant regulations and ethical guidelines. CPAP‐related symptoms collection was conducted via telephone by Ms. Shi‐Shan Pan, a certified sleep technologist and case manager for sleep disorders. Semistructured telephone interviews were carried out during the initial phase of CPAP use to gather information on patients’ experiences, including their concerns and commonly reported discomfort related to CPAP therapy. In general, patients who agreed to try CPAP received follow‐up phone calls to monitor symptoms of discomfort and address issues related to device settings, such as pressure or temperature, on Days 3 and 7, as well as one day prior to their follow‐up visit. If any machine settings were adjusted by the case manager, an additional follow‐up call was made the following day to assess the patient’s response. Discomfort was documented through open‐ended questions (see Supporting File 2).

### 2.7. Statistical Analysis

Participant demographics were summarized using descriptive statistics (means, standard deviation, and percentages). Student’s *t*‐tests or chi‐square tests examined differences in CPAP adherence relative to demographics and symptoms. Symptom variables, categorized into main domains and subdomains, were developed from qualitative data and validated via a Delphi process. To appropriately address within‐patient clustering resulting from repeated symptom assessments, GEEs were used for all inferential analyses examining associations between symptoms and CPAP adherence.

## 3. Results

### 3.1. Demographic Data of the Participants

A total of 121 patients diagnosed with OSA were enrolled through a sleep specialist clinic. All participants consented to initiate a CPAP therapy trial. The average age of 121 participants was 50.78 years, with males comprising 84.3% of the subjects. The mean BMI and AHI exceeded normal ranges, at 28.05 kg/m^2^ and 51.16 events/hour, respectively. The mean daytime sleepiness score, as measured by the ESS, was 9.31. Most participants had an education level of college or higher (57.0%). The participants had an average of 1.71 ± 1.43 comorbidities, with the three most common chronic conditions being hypertension (40.5%), gastroesophageal reflux disease (36.4%), and hyperlipidemia (22.3%). Among the participants, 18.2% reported smoking and 28.9% reported alcohol consumption. More than half reported exercising (60.3%) and had a habit of taking an afternoon nap (66.1%). Only seven participants (5.8%) had previously undergone surgeries related to OSA treatment (see Table 1).

**TABLE 1:** Demographic data of the participants.

Variables	Overall (*N* = 121)		Poor adherence (*n* = 48, 39.7%)		Good adherence (*n* = 73, 60.3%)		*p* value
	*n*	%	*n*	%	*n*	%	
Age (years)							0.393
≤ 40	32	26.4	15	31.3	17	23.3	
41–60	56	46.3	21	43.8	35	47.9	
> 61	33	27.3	12	25.0	21	28.8	
Mean, SD	50.78, 13.13		49.13, 13.11		51.86, 13.13		0.264
Sex							0.176
Female	19	15.7	5	10.4	14	19.2	
Male	102	84.3	43	89.6	59	80.3	
BMI (kg/m^2^)							0.145
Mean, SD	28.05, 4.45		28.77, 4.61		27.56, 4.31		
AHI (events/hour)							0.947
Mean, SD	51.16, 25.22		50.97, 25.00		51.28, 25.53		
ESS							
Mean, SD	9.31, 4.82		8.94, 4.33		9.55, 5.12		0.502
Level of education							0.610
High school and below	52	43.0	22	45.8	30	41.1	
College/university and above	69	57.0	26	54.2	43	58.9	
Comorbidity							
Hypertension	49	40.5	21	43.8	28	38.4	0.558
Hyperlipidemia	27	22.3	12	25.0	15	20.5	0.569
Hyperglycemia	13	10.7	6	12.5	7	9.6	0.616
DM	13	10.7	5	10.4	8	11.0	0.926
CVD	19	15.7	6	12.5	13	17.8	0.437
Parkinson’s disease	0	0.0	0	0.0	0	0.0	
CVA	3	2.5	1	2.1	2	2.7	0.822
Mild cognitive impairment	1	0.8	0	0.0	1	1.4	0.420
COPD	1	0.8	1	2.1	0	0.0	0.322
Asthma	6	5.0	3	6.3	3	4.1	0.599
Malignant neoplasm	3	2.5	1	2.1	2	2.7	0.822
Mental disorders	5	4.1	1	2.1	4	5.5	0.363
GERD	44	36.4	21	43.8	23	31.5	0.180
Others	23	19.0	9	18.8	14	19.2	0.954
Mean, SD	1.71, 1.43		1.81, 1.58		1.64, 1.33		0.529
Smoking	22	18.2	11	22.9	11	15.1	0.294
Alcohol consumption	35	28.9	18	37.5	17	23.3	0.104
Exercise	73	60.3	27	56.3	46	63.0	0.461
Afternoon nap	80	66.1	33	68.8	47	64.4	0.623
Ever experienced OSA‐related surgery	7	5.8	5	10.4	2	2.7	0.119
CPAP usage (%)							
Mean, SD	64.78, 32.14		30.23, 22.15		87.49, 9.06		**< 0.001**

*Note:* Bold font indicates results that are statistically significant. GERD, gastroesophageal reflux; CVA, cerebrovascular accident; CVD, cardiovascular disease.

Abbreviations: AHI, apnea and hypopnea index; BMI, body mass index; COPD, chronic obstructive pulmonary disease; DM, diabetes mellitus; OSA, obstructive sleep apnea; SD, standard deviation.

### 3.2. Adherence to CPAP

During the CPAP trial period, the average CPAP usage was 64.78% (±32.14), with 60.3% of the participants demonstrating good adherence. A total of 73 participants met these criteria, with an average adherence rate of 87.49% ± 9.06. There were no statistically significant differences in demographic characteristics between patients with good and poor CPAP adherence (see Table 1).

### 3.3. Follow‐Up Treatment for Patients With Moderate and Severe OSA

**FIGURE 1:**
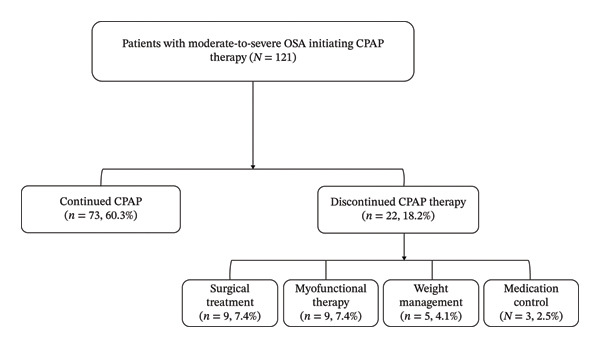
Flow diagram of participant disposition and CPAP treatment decisions during the initial therapy period.

After the 2‐week trial, 73 participants (60.3%) continued using CPAP. Among those who discontinued CPAP, alternative treatments included standard follow‐up (without intervention) (18.2%), surgical treatment (7.4%), myofunctional therapy (6.6%), weight management (5.0%), and medication control (2.5%) (see Figure 1).

### 3.4. Main and Subdomains of Symptoms and Specific Patients’ Symptoms During the 2‐Week CPAP Application

Among the 121 participants, a total of 430 symptom observations were recorded, representing repeated reports of CPAP‐related complaints across three interview time points. These complaints were subsequently classified into 30 distinct symptom categories (see Supporting Table 1) and further grouped into four major domains: physiological, psychological, CPAP interface–related (discomfort resulting from direct contact between the machine and the body), and CPAP device–related (issues arising from the equipment itself) (see Figure 2(a)). A detailed breakdown is as follows:

FIGURE 2Domains, subdomains, and specific patients’ symptoms during the 2‐week CPAP application period (*N* observations = 430). (a) CPAP‐related subdomains grouped by main domain category; (b) detailed CPAP‐related symptoms ranked by frequency during the 2‐week CPAP trial period. Note: percentages were calculated based on the total of 430 symptom observations, representing repeated reports collected across three interview time points.

**Figure:**
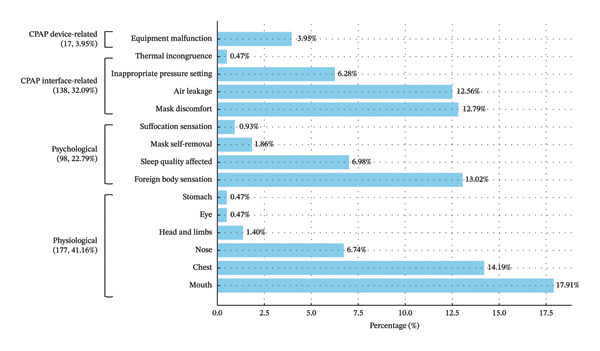
(a)

**Figure:**
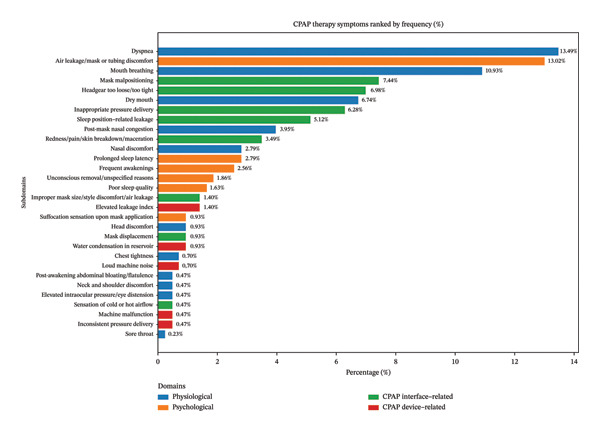
(b)

#### 3.4.1. Physiological

Physiological symptoms were the most commonly reported discomfort following CPAP use (approximately 44.16%). It was further divided into discomfort involving the mouth, eyes, nose, head and limbs, chest, and gastrointestinal system. Among these, discomfort in the mouth (17.91%) and chest (14.19%) regions was most prevalent (see Figure 2(a)). The most frequently reported physiological complaint was dyspnea during CPAP use (13.49%), followed by mouth breathing (10.93%) and dry mouth (6.74%) (see Figure 2(b)).

#### 3.4.2. Psychological

Approximately 22.79% of participants reported experiencing psychological discomfort. The most frequently reported symptoms included foreign body sensations (13.02%) and sleep quality–related disturbances (6.98%) (see Figure 2(a)). The most common psychological complaints were foreign body sensation caused by air leakage, mask, or tubing (13.02%), followed by prolonged sleep onset latency (2.79%), frequent nocturnal awakenings (2.56%), and unconscious mask removal during sleep (1.86%) (see Figure 2(b)).

#### 3.4.3. CPAP Interface–Related

Approximately 32.09% of the participants experienced discomfort due to the interface between the CPAP machine and their body. Mask discomfort (12.80%) and air leakage (12.56%) were the most common complaints in this category (see Figure 2(a)). More frequently, interface‐related issues included mask malpositioning (7.44%), headgear that was either too tight or too loose (6.98%), and inappropriate pressure settings (6.28%) (see Figure 2(b)).

#### 3.4.4. CPAP Device–Related

Approximately 3.95% of the participants reported being disturbed by factors related to the CPAP equipment itself (see Figure 2(a)), including an elevated machine leakage index (1.40%), water condensation in the humidifier reservoir (0.93%), or excessive machine noise (0.70%) (see Figure 2(b)).

### 3.5. Domain‐Level Symptoms and CPAP Adherence

Furthermore, we compared mean differences in CPAP adherence outcomes between the good‐ and poor‐adherence groups according to reported symptom domains. Among the 4 main domains and 15 subdomains, we found that psychological factors were significantly associated with adherence (*p* = 0.025), with patients in the poor adherence group reporting higher psychological symptom scores (mean = 0.31) than those in the good adherence group did (mean = 0.20). Interestingly, the overall CPAP interface symptom score was higher in the good adherence group (*p* = 0.023). Symptoms related to the CPAP interface, including air leakage (*p* = 0.011) and mask discomfort (*p* = 0.047), were significantly more common in the good adherence group (see Table 2).

**TABLE 2:** CPAP adherence outcomes between good‐ and poor‐adherence groups according to reported symptom domains (*N* observations = 430).

Variables	Poor adherence	Good adherence	*p* value
	Mean, SD		
Physiological	0.46, 0.58	0.47, 0.63	0.882
Mouth	0.18, 0.44	0.23, 0.51	0.293
Eye	0.01, 0.10	0.00, 0.00	0.158
Nose	0.07, 0.25	0.08, 0.27	0.672
Head and limbs	0.01, 0.07	0.02, 0.15	0.136
Chest	0.19, 0.45	0.14, 0.41	0.265
Stomach	0.01, 0.10	0.00, 0.00	0.158
Psychological	0.31, 0.55	0.20, 0.44	**0.025**
Sleep quality affected	0.08, 0.33	0.05, 0.28	0.355
Foreign body sensation	0.19, 0.45	0.12, 0.34	0.062
Mask self‐removal	0.02, 0.15	0.02, 0.13	0.777
Suffocation sensation	0.02, 0.13	0.00, 0.07	0.254
CPAP interface–related	0.27, 0.49	0.38, 0.56	**0.023**
Air leakage	0.08, 0.30	0.18, 0.44	**0.011**
Mask discomfort	0.09, 0.30	0.16, 0.40	**0.047**
Inappropriate pressure setting	0.09, 0.33	0.04, 0.20	0.057
Thermal incongruence	0.00, 0.00	0.01, 0.09	0.158
CPAP device–related	0.03, 0.18	0.06, 0.29	0.275
Equipment malfunction	0.03, 0.18	0.06, 0.29	0.275

*Note:* Bold font indicates results that are statistically significant.

### 3.6. The Impact of Domain‐Level Symptoms on CPAP Adherence

A GEE analysis was conducted in Model 1 to examine the association between symptoms and CPAP adherence. No statistically significant associations were observed for most demographic or clinical variables. However, among the four major domains of symptoms analyzed, only psychological discomfort had a statistically significant adverse effect on CPAP adherence. Participants who reported psychologically related complaints, such as disturbed sleep quality, foreign body sensation, or mask‐related anxiety, exhibited 7.50% lower adherence compared to those without such complaints (95% CI: −14.95–−0.05, *p* = 0.049). In contrast, physiological discomfort (e.g., dry mouth, chest discomfort), CPAP interface–related issues (e.g., air leakage, mask discomfort), and CPAP device–related problems did not significantly affect adherence (see Table 3).

**TABLE 3:** Generalized equation analysis of the impact of main domains and subdomains on CPAP adherence.

Variables	Percentage of CPAP adherence (*N* observations = 430)		*p* value
	Adjusted coef.	95% CI	
Model 1			
Age (years)	−0.31	−1.22, 0.58	0.489
Sex (reference: female)	−12.26	−30.87, 6.35	0.197
AHI (events/minute)	−0.06	−0.48, 0.36	0.783
BMI (kg/m^2^)	−0.16	−2.91, 2.60	0.912
Education level (reference: high school and below)	6.85	−13.45, 27.14	0.508
Number of comorbidities	−0.69	−5.56, 4.17	0.781
Alcohol consumption	−14.26	−30.03, 1.52	0.076
Smoke	−2.85	−24.28, 18.57	0.794
Exercise	2.96	−13.31, 19.22	0.722
Afternoon nap	3.33	−14.93, 21.59	0.721
Ever experienced OSA‐related surgery	−8.95	−38.49, 20.60	0.553
The main domains of symptoms			
Physiological	2.50	−4.46, 9.46	0.481
Psychological	−7.50	−14.95, −0.05	**0.049**
CPAP interface–related	7.28	−1.22, 15.77	0.093
CPAP device–related	7.45	−2.86, 17.75	0.157
Model 2^a^			
Subdomains of symptoms			
Mouth	3.51	−6.83, 13.85	0.506
Eye	−5.21	−27.79, 17.38	0.651
Nose	11.35	−3.56, 26.25	0.136
Head and limbs	24.03	−5.34, 53.39	0.109
Chest	−1.03	−9.22, 7.16	0.805
Gastrointestinal	12.09	−12.43, 36.61	0.334
Sleep quality affected	−2.69	−12.08, 6.70	0.575
Foreign body sensation	−5.94	−17.36, 5.49	0.308
Mask self‐removal	−11.56	−36.36, 13.23	0.361
Suffocation sensation	−37.14	−68.03, −6.25	**0.018**
Air leakage	11.01	−1.06, 23.07	0.074
Mask discomfort	12.89	2.92, 22.87	**0.011**
Inappropriate pressure delivery	−6.77	−18.14, 4.60	0.243
Thermal incongruence	8.83	−11.33, 28.99	0.391
Equipment malfunction	7.89	−3.17, 18.96	0.162

*Note:* Bold font indicates results that are statistically significant.

^a^Denotes adjusted covariates, including age, sex, AHI, BMI, education level, number of comorbidities, alcohol consumption, smoking status, exercise, and prior OSA‐related surgery.

Model 2 further investigated the influence of individual subdomains on CPAP adherence after adjusting for demographic data. Among the various subdomains analyzed, patients who reported suffocation sensations during CPAP use exhibited a 37.14% reduction in adherence (95% CI: −68.03–−6.25, *p* = 0.018) compared to those without such symptoms. However, those who reported mask discomfort, including skin redness/pain, mask displacement, headgear too loose/tight, or improper mask size, demonstrated 12.89% greater adherence (95% CI: 2.92–22.87, *p* = 0.011) (see Table 3).

## 4. Discussion

In this prospective mixed‐methods study of 121 adults with moderate‐to‐severe OSA undergoing an initial CPAP trial, we systematically characterized CPAP‐related symptom experiences and examined their associations with early CPAP adherence. During the 2‐week trial period, a total of 430 symptom observations were identified and categorized into four major domains: physiological, psychological, CPAP interface–related, and CPAP device–related symptoms. Physiological symptoms were the most frequently reported, followed by interface‐related and psychological symptoms, whereas device‐related problems were relatively uncommon. Importantly, only psychological symptoms were consistently associated with reduced CPAP adherence, with participants reporting psychological discomfort exhibiting significantly lower adherence. In contrast, physiological and CPAP device–related symptoms were not associated with adherence, and CPAP interface–related symptoms—particularly mask discomfort and air leakage—were more frequently reported among participants with good adherence. These findings highlight the distinct and differential roles of symptom domains in shaping early CPAP adherence behavior.

In our study, we identified four major domains of CPAP‐related symptoms: physiological, psychological, CPAP interface–related, and CPAP device–related, and examined their associations with early CPAP adherence. These findings align with those of the foundational review by Ghadiri and Grunstein, who proposed a taxonomy of CPAP‐related side effects based on clinical symptoms and patient‐reported outcomes. Their framework emphasized pressure‐related physiological adaptations and user‒machine interface issues as the most common adverse experiences [6]. Our study identified two additional symptom domains that warrant attention. The first is psychological symptoms, including sleep disturbance, foreign body sensation, and claustrophobia, which were shown to significantly affect adherence in our cohort. The second involves complaints related to CPAP performance, such as equipment malfunction or inconsistent pressure delivery, as a distinct symptom domain. By incorporating these domains, our findings underscore the multifaceted nature of CPAP‐related symptom experiences during the early treatment adaptation phase and suggest that both technical and psychosocial factors warrant attention when supporting patients initiating CPAP therapy.

Our study revealed that psychological discomfort was the only primary domain significantly associated with reduced CPAP adherence during the initial trial period. This factor was associated with a 7.50% decrease in CPAP usage. In particular, claustrophobic sensations showed as a key psychological barrier to continued CPAP use. These findings are consistent with previous research. Wild et al. (2004) conducted a prospective study involving 119 OSA patients and demonstrated that psychological factors were significant predictors of CPAP adherence, as measured by validated psychological questionnaires [14]. Similarly, in a cross‐sectional study, Poulet et al. reported that emotional responses to CPAP therapy strongly influenced patients′ early experiences and subsequent adherence [15]. Our results also further support earlier findings that claustrophobia [6] and difficulty initiating sleep [8] are critical barriers to consistent CPAP use. These findings add to a growing body of evidence indicating that subjective psychological responses, rather than physical or technical factors, play a central role in shaping patients’ acceptance and early use of CPAP therapy. They also highlight the importance of early psychosocial assessment and support at the beginning of CPAP therapy. Interventions such as motivational interviewing [16], cognitive‒behavioral therapy for insomnia (CBT‐I) [17], or brief CPAP desensitization protocols [18] for claustrophobia may be valuable in mitigating psychological barriers during the early CPAP adaptation phase.

In our study, physiological symptoms were the most commonly reported complaints following CPAP use, followed by CPAP interface–related issues. This pattern is consistent with the review by Ghadiri and Grunstein, which highlighted physiological side effects and interface‐related discomfort as the most frequently reported problems among CPAP users. Similarly, Park et al. reported that 21.7% of CPAP users experienced chest discomfort and that 13.1% complained of dry mouth, reinforcing the prominence of physiological complaints during therapy. In addition, our findings align with previous studies indicating that discomfort caused by a mismatch between facial anatomy and mask design [19], as well as persistent air leakage [20], are key contributors to poor user experience and therapy interruption. These observations underscore the importance of individualized mask fitting, proactive troubleshooting, and ongoing symptom management to enhance comfort and support patients initiating CPAP therapy.

Interestingly, our findings showed that patients who reported CPAP interface–related problems, such as air leakage and mask discomfort, were more likely to demonstrate better CPAP adherence. Previous studies have similarly noted that appropriate guidance from the medical team, selecting a well‐fitted and suitable mask, can enhance adherence by minimizing discomfort and improving the overall user experience [20]. Similarly, Genta et al. noted that interface‐related side effects are commonly reported by CPAP users; however, actively addressing these issues, particularly during the first week of therapy, can significantly improve adherence to CPAP treatment [21]. These findings imply that interface‐related complaints should not be viewed purely as adverse outcomes but rather as early signals that can prompt clinicians to provide timely interventions, such as mask refitting, humidification adjustments, or reassurance. Addressing these symptoms effectively may not only alleviate discomfort but also support continued CPAP behavior during the early adaptation period.

This study has some limitations that should be acknowledged. First, it was conducted at a single tertiary medical center in southern Taiwan, which may limit the generalizability of the findings to broader or more diverse populations. Second, follow‐up was limited to a short CPAP trial period, which precludes direct conclusions regarding long‐term adherence and clinical outcomes. Accordingly, the adherence measures in this study reflect early treatment behavior rather than established CPAP use and should not be interpreted as direct evidence of sustained long‐term adherence.

## 5. Conclusions

This prospective mixed‐methods study provides a structured and comprehensive evaluation of CPAP‐related adverse symptoms and their associations with early treatment adherence among patients with moderate‐to‐severe OSA. These findings highlight psychological discomfort, such as foreign body sensations, disturbed sleep quality, and claustrophobic feelings, as the only symptom domain significantly associated with reduced initial CPAP adherence. Moreover, patients who reported interface‐related issues such as mask discomfort and air leakage showed better adherence, suggesting that early detection and clinical management of such symptoms may support continued CPAP use in clinical practice. These results emphasize the importance of early psychosocial assessment and individualized support during the initial stages of therapy.

**Nomenclature**
AHI: Apnea‒hypopnea index
BMI: Body mass index
CPAP: Continuous positive airway pressure
GEE: Generalized estimating equation
OSA: Obstructive sleep apnea
PSG: Polysomnography

## Author Contributions

Shin‐Shan Pan, Cheng‐Man Ng, Cheng‐Yu Lin, Yen‐Hsu Chen, and Yen‐Chin Chen were responsible for the study design. Cristina Frange, Shin‐Shan Pan, Cheng‐Man Ng, and Yen‐Chin Chen completed the data analysis and manuscript preparation.

## Funding

This study was funded by the National Science and Technology Council, NSTC 112‐2628‐B‐006‐010‐MY3; Geriatric Medicine Translational Research Group.

## Disclosure

Cristina Frange, Cheng‐Yu Lin, Yen‐Hsu Chen, and Yen‐Chin Chen approved the final manuscript.

## Ethics Statement

This study was approved by the institutional review board of the National Cheng Kung University Hospital (IRB No. A‐ER‐111–531). All patients provided written informed consent after being notified of the study procedures.

## Conflicts of Interest

The authors declare no conflicts of interest.

## Supporting Information

Additional supporting information can be found online in the Supporting Information section.

## Supporting information


**Supporting Information 1** Supporting File S1. The Reporting of Observational Studies in Epidemiology (STROBE) guidelines.

**Supporting Information 2** Supporting File S2. Initial CPAP Phone Interview Guide.

**Supporting Information 3** Supporting Table S1. Domains, subdomains, and symptom categories of CPAP therapy–related symptoms during the 2 week CPAP application period (*N* observations = 430).

## Data Availability

The data that support the findings of this study are available upon request from the corresponding author (Dr. Yen‐Chin Chen).
