# Supplementary material for: Beyond the Mask―Psychological Discomfort as a Predictor of Early CPAP Nonadherence in Moderate‐to‐Severe OSA Patients: A Prospective Mixed‐Methods Study
Source: Nurs Res Pract. 2026 Apr 6;2026:9630236. doi: 10.1155/nrp/9630236 (PMC13053947; doi:10.1155/nrp/9630236)
Supplement: Supplementary file 2 — Supporting Information 2 Supporting File S2. Initial CPAP Phone Interview Guide. [file NRP-2026-9630236-s003.docx]

Supplementary File S1. Initial CPAP Phone Interview Guide

**Opening**

Hello Mr./Ms. [Last Name], this is XXX from the Sleep Disorder Care Team at National Cheng Kung University Hospital. Since you’ve been using a CPAP machine for almost 3 months or 1 year, we’d like to check in and understand how you’re doing. Your feedback helps us identify any discomfort or difficulties you may be experiencing with CPAP, and we can provide suggestions or support accordingly. Would it be okay to speak with you for about 15–20 minutes?

☐ Yes / ☐ No

P.S. If now is not a good time, may I ask when would be a better time to contact you again?

------------------------------------------------------------------------------------------------------

**Best time to call back:**

If the patient says **No**:

Thank you, and sorry to have disturbed you. If you have any questions later on, feel free to call this number.

If the patient says **Yes**:

Thank you for your cooperation. If at any point you'd like to pause, just let me know. Let’s confirm a few things first.

May I confirm if it’s convenient to speak now?
☐ Yes / ☐ No

Can you hear me clearly?
☐ Yes / ☐ No

Thank you. Let’s begin.

1. Have you noticed any changes (good or bad) since using the CPAP machine? How has it affected you?
   ANS:
2. Have you encountered any uncomfortable symptoms while using the CPAP?
3. So far, do you feel that CPAP use has been helpful to you?

☐ Yes / ☐ No

**Closing**

Mr./Ms. [Last Name], are you feeling tired? You’ve done great—please take a short break. (Small talk if appropriate.) Feel free to reach out if anything comes up. Goodbye!
