# Supplementary material for: Beyond the Mask―Psychological Discomfort as a Predictor of Early CPAP Nonadherence in Moderate‐to‐Severe OSA Patients: A Prospective Mixed‐Methods Study
Source: Nurs Res Pract. 2026 Apr 6;2026:9630236. doi: 10.1155/nrp/9630236 (PMC13053947; doi:10.1155/nrp/9630236)
Supplement: Supplementary file 3 — Supporting Information 3 Supporting Table S1. Domains, subdomains, and symptom categories of CPAP therapy–related symptoms during the 2 week CPAP application period (N observations = 430). [file NRP-2026-9630236-s001.docx]

Supplementary Table 1. Domains, subdomains, and specific patients' symptoms during 2-week CPAP application period (N observations = 430)

| **Domain** | **Subdomain** | **Sub-domains (%)** | **Symptoms category** | **n** | **%** |
| --- | --- | --- | --- | --- | --- |
| Physiological (177, 41.16%)^¶^ | Mouth | 17.91 | Dry mouth | 29 | 6.74 |
|  |  |  | Mouth breathing | 47 | 10.93 |
|  |  |  | Sore throat | 1 | 0.23 |
|  | Eye | 0.47 | Elevated intraocular pressure/ eye distension | 2 | 0.47 |
|  | Nose | 6.74 | Nasal discomfort | 12 | 2.79 |
|  |  |  | Post-mask nasal congestion | 17 | 3.95 |
|  | Head and limbs | 1.40 | Head discomfort | 4 | 0.93 |
|  |  |  | Neck and shoulder discomfort | 2 | 0.47 |
|  | Chest | 14.19 | Dyspnea | 58 | 13.49 |
|  |  |  | Chest tightness | 3 | 0.70 |
|  | Stomach | 0.47 | Post-awakening abdominal bloating/ flatulence | 2 | 0.47 |
| Psychological (98, 22.79%)^¶^ | Sleep quality affected | 6.98 | Frequent awakenings | 11 | 2.56 |
|  |  |  | Prolonged sleep latency | 12 | 2.79 |
|  |  |  | Poor sleep quality | 7 | 1.63 |
|  | Foreign body sensation | 13.02 | Air leakage/ mask or tubing discomfort | 56 | 13.02 |
|  | Mask self-removal | 1.86 | Unconscious removal/ unspecified reasons | 8 | 1.86 |
|  | Suffocation sensation | 0.93 | Suffocation sensation upon mask application | 4 | 0.93 |
| Ventilator interface related (138, 32.09%)^¶^ | Air leakage | 12.56 | Sleep position related | 22 | 5.12 |
|  |  |  | Malpositioning of the mask | 32 | 7.44 |
|  | Mask discomfort | 12.79 | Redness/ pain/ skin Breakdowns/ maceration | 15 | 3.49 |
|  |  |  | Mask displacement | 4 | 0.93 |
|  |  |  | Headgear too loose/ tight | 30 | 6.98 |
|  |  |  | Improper mask size/ mask style discomfort/ air leakage | 6 | 1.40 |
|  | Inappropriate pressure delivery | 6.28 | Occurring at mask-on | 19 | 4.42 |
|  |  |  | After sleep onset | 3 | 0.70 |
|  |  |  | Leak-related | 5 | 1.16 |
|  | Thermal incongruence | 0.47 | Sensation of cold or hot air | 2 | 0.47 |
| CPAP device related (17, 3.95%)^¶^ | Equipment malfunction | 3.95 | Loud machine noise | 3 | 0.70 |
|  |  |  | Water condensation in reservoir | 4 | 0.93 |
|  |  |  | Machine malfunction | 2 | 0.47 |
|  |  |  | Inconsistent pressure delivery | 2 | 0.47 |
|  |  |  | Elevated leakage index | 6 | 1.40 |

Note: ¶Percentages are calculated using the total number of symptom observations (N = 430), which represent repeated reports across three interview time points.

CPAP, continuous positive airway pressure.
